# Seventeen Years of an Antibiotic Stewardship Programme: Trends in Antibiotic Prescribing and Gram-Negative Bacilli Susceptibility at a Quaternary Healthcare Institution

**DOI:** 10.3390/antibiotics14121239

**Published:** 2025-12-08

**Authors:** Yvonne Peijun Zhou, Shimin Jasmine Chung, Winnie Hui Ling Lee, Yibo Wang, Shena Yun Chun Lim, Yen Ee Tan, Andrea Lay Hoon Kwa

**Affiliations:** 1 Division of Pharmacy, Singapore General Hospital, Outram Road, Singapore 169608, Singapore; winnie.lee.h.l@sgh.com.sg (W.H.L.L.); wang.yibo@sgh.com.sg (Y.W.); andrea.kwa.l.h@sgh.com.sg (A.L.H.K.); 2 Department of Infectious Diseases, Singapore General Hospital, Outram Road, Singapore 169608, Singapore; 3 Department of Microbiology, Singapore General Hospital, Outram Road, Singapore 169608, Singapore; tan.yen.ee@singhealth.com.sg; 4 SingHealth Duke-NUS Medicine Academic Clinical Programme, 8 College Road, Singapore 169857, Singapore; 5 Emerging Infectious Disease Program, Duke-NUS Medical School, 8 College Road, Singapore 169857, Singapore

**Keywords:** antibiotic stewardship, antibiotic appropriateness, antibiotic consumption, antibiogram, Gram-negative bacilli susceptibility, resistance

## Abstract

**Background/objectives:** Studies evaluating the longitudinal impact (beyond a decade) of antibiotic stewardship programs (ASPs) on the volume/quality of antibiotic prescriptions, as well as the impact on antibiotic resistance, are lacking. Since 2008, the ASP at Singapore General Hospital has implemented various strategies in the following phases: (1) initiation, (2) expansion, (3) optimisation, and (4) innovation. In this study, we aim to evaluate the volume/quality of antibiotic prescribing and susceptibility trends of clinically significant Gram-negative bacilli (GNBs), along with the evolution of ASP strategies over time. **Methods:** We conducted a single-centre, retrospective observational study from 2011 to 2024. Antibiotic consumption, appropriateness, and susceptibility trends of six GNBs to seven commonly used antibiotics were analysed using the *Kendall tau* test to identify potential monotonic trends based on aggregated rather than patient-level data. **Results:** We demonstrated sustained improvement in appropriateness of seven broad-spectrum IV antibiotics, accompanied by significant reductions in IV ciprofloxacin, cefepime, and ertapenem use (*p* < 0.05). Hospital-wide susceptibility of six GNBs to all evaluated antibiotics improved significantly (*p* < 0.05), except for *E. coli*’s susceptibility to ertapenem and *Enterobacterales*’s susceptibility to ciprofloxacin. **Conclusions:** With an evolving, multi-pronged stewardship approach, antibiotic prescribing and GNB susceptibility to most antibiotics have improved. In a rapidly evolving healthcare landscape, ASPs must remain agile, continually refining priorities and employing innovative strategies.

## 1. Introduction

Antibiotic stewardship programs (ASPs) are advocated in all healthcare institutions to combat rising bacterial resistance [1,2]. Globally, ASP implementation has reduced antibiotic consumption and, hence, improved Gram-negative bacillus (GNB) susceptibility rates, including *P. aeruginosa* and extended-spectrum β-lactamase (ESBL)-producing *Enterobacterales* towards broad-spectrum antibiotics across various healthcare settings [3,4,5,6,7,8,9]. Despite the growing body of literature on ASPs, there remains a notable scarcity of longitudinal studies extending beyond a decade evaluating sustained impact of ASPs on the volume and quality of antibiotic prescribing, along with antibiotic susceptibility trends.

**Figure 1:**
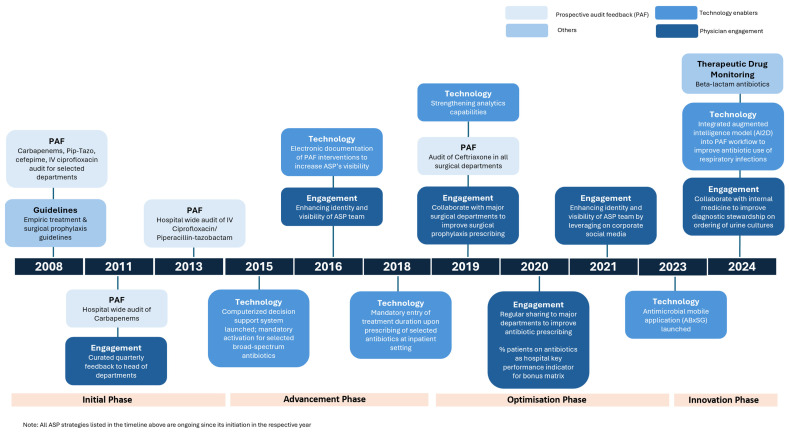
Antibiotic stewardship strategies implemented at Singapore General Hospital from 2008 to 2024.

At Singapore General Hospital (SGH), an 1800-bed quaternary care hospital in Singapore with diverse medical and surgical specialties (including haematology, oncology, and transplant), the ASP team, comprising infectious diseases physicians and clinical pharmacists, was officially established in 2008. Since its inception, multi-pronged antibiotic stewardship strategies have evolved over 17 years to address the gaps in prescribing patterns. The growth of the ASP can be mainly categorised into the following four phases: (1) initiation, (2) advancement, (3) optimisation, and (4) innovation [Table 1, Figure 1].

### 1.1. Initiation Phase (2008–2014)

Our ASP prioritised preserving GNB susceptibility over Gram-positive cocci (GPCs) due to the greater clinical and therapeutic challenges posed by GNBs, particularly given the low susceptibility rates observed in GNBs such as *Acinetobacter baumannii* and *Pseudomonas aeruginosa* at our institution. Hence, in the initiation phase, our priority was to promote the appropriate use of carbapenems (ertapenem/meropenem) and anti-pseudomonal agents (IV ciprofloxacin/piperacillin–tazobactam) through hospital-wide prospective audit and feedback (PAF). When antibiotics were deemed inappropriate based on institution guidelines, ASP pharmacists/physicians made recommendations to the attending physicians, which included antibiotic discontinuation, de-escalation, escalation, intravenous (IV)-to-oral switch, dose adjustments, and formal infectious diseases consultation. With PAF, we demonstrated shorter length of stay and lower 14-day re-infection and infection-related readmission rates in cases where ASP recommendations were adopted [10,11,12].

### 1.2. Advancement Phase (2015–2018)

Next, we leveraged technology to influence antibiotic prescribing. In 2015, a computerised decision support system (CDSS) was integrated into the electronic medical records and made mandatory for select broad-spectrum antibiotics. In 2018, the entry of treatment duration during electronic prescribing was made compulsory for select antibiotics to enhance physician accountability. Concurrently, we strengthened the visibility and branding of the ASP team, positioning the ASP team as collaborative partners in patient care rather than “law enforcers”. For example, ASP pharmacists participated in ward rounds in high antibiotic use departments to engage and support prescribers.

### 1.3. Optimisation Phase (2019–2023)

Serial in-house studies revealed a high prevalence of antibiotic use (~50% of admitted patients received antibiotics, substantially higher than in healthcare systems across Europe and North America (11–33%) [13]), and our strategies pivoted. We expanded in scope from solely infection management to include surgical prophylaxis, targeting instances where narrower-spectrum agents may be misused. For example, PAF was expanded to ceftriaxone across surgical wards, accompanied by collaborative efforts with surgical departments to shorten post-surgery prophylaxis [14].

Strong support from hospital senior leadership facilitated key stewardship initiatives, including enhancing analytical capabilities, fostering collaboration between ASP and clinical departments, promoting ASP awareness hospital-wide, and linking antibiotic stewardship metrics to hospital incentives.

### 1.4. Innovation Phase (2023 to Date)

In the era of digital transformation, our ASP approach is novel in that we enhanced our digital capabilities to advance antibiotic stewardship by leveraging multiple aspects of advanced digital technology within a single quaternary healthcare institution. In 2023, we became the first in Singapore to launch an antibiotic mobile application, ABxSG, as an antibiotic stewardship tool to empower physicians and pharmacists to optimise antibiotic use [15]. Simultaneously, we were also the first in the nation to develop and implement a machine learning model, AI^2^D (Augmented Intelligence in Infectious Diseases), which predicts the likelihood of bacterial lower respiratory tract infections. Integrating AI^2^D into our PAF workflow improved PAF effectiveness [16].

In the arena of precision medicine, β-lactam therapeutic drug monitoring (TDM) to guide individualised drug dosing was introduced for critically ill patients to optimise outcomes and curb resistance emergence [17,18].

### 1.5. Study Objectives

The primary objective of this study is to evaluate the volume (i.e., consumption) and quality (i.e., appropriateness) of antibiotic prescribing along with the evolution of ASP strategies. The secondary objective is to examine the longitudinal susceptibility trends of common Gram-negative bacilli (GNBs) over time.

## 2. Methods

### 2.1. Data Collection

Data on IV antibiotic consumption (available from 2011 onwards) was obtained from the hospital data warehouse and converted into defined daily doses per 1000 inpatient days according to the definitions by the 2024 World Health Organization (WHO) Anatomical Therapeutic Chemical (ATC) classification system [19]. Appropriate use of antibiotics (i.e., IV ciprofloxacin, ceftriaxone, piperacillin–tazobactam, ertapenem, and meropenem) under PAF were obtained from SGH’s antibiotic stewardship electronic audit system. The appropriateness of antibiotic use was evaluated based on antibiotic indication, choice, duration, route, and dose upon antibiotic initiation and discontinuation.

Susceptibility data, which was only available from 2011 onwards (and 2014 onwards for carbapenems), was derived from the antibiogram constructed by the institution microbiology laboratory based on definitions by the Clinical & Laboratory Standards Institute (CLSI) [20]. The antibiogram was compiled annually using clinical samples and for GNBs with ≥30 isolates, including only the first isolate per patient within the same calendar year. The antibiogram of 6 clinically significant GNBs [i.e., *Acinetobacter baumannii*, *Pseudomonas aeruginosa*, *E. coli*, *Klebsiella* spp. (excluding *Klebsiella aerogenes*), *Enterobacter* spp./*Klebsiella aerogenes*, *Citrobacter freundii]* towards the commonly used antibiotics (ciprofloxacin, ceftriaxone, amoxicillin–clavulanate, cefepime, piperacillin–tazobactam, ertapenem, and meropenem) was generated. *Klebsiella aerogenes* was classified alongside *Enterobacter* spp. as it was formerly known as *Enterobacter aerogenes*. Susceptibility was interpreted based on CLSI breakpoints that were recommended for the respective years. Notably, there was a reduction in susceptible breakpoint for piperacillin–tazobactam (≤64/4 to ≤16/4 μg/mL) for *P. aeruginosa* and *Enterobacterales* (≤16/4 to ≤8/4 μg/mL) in 2012 and 2022, respectively [21,22]. The susceptible breakpoint was also reduced for cefepime (≤8 to ≤2 μg/mL) towards *Enterobacterales* in 2014 [23].

### 2.2. Statistical Analysis

Statistical tests were conducted using SPSS (Version 26.0, IBM Corp., Armonk, NY, USA). The trend of antibiotic consumption, appropriateness, and susceptibility data over time were analysed using the *Kendall tau* test. *Kendall tau* was employed because the analysis was exploratory and aimed to identify monotonic patterns at the aggregated level (instead of the patient level) without inferring causality. Continuous data are presented as the median (interquartile range), unless stated otherwise.

## 3. Results

### 3.1. Antibiotic Consumption

**Figure 2:**
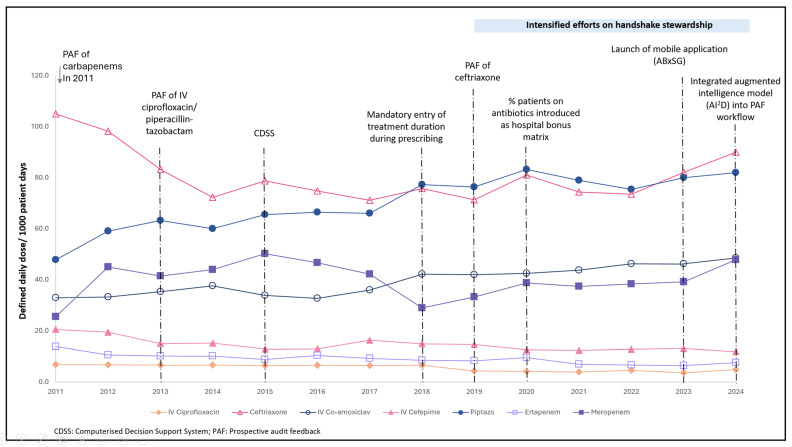
Consumption of antibiotics (defined daily doses/1000 patient-days) along with various antibiotic stewardship strategies from 2011 to 2024.

From 2011 to 2024, a significant reduction in consumption was shown for IV ciprofloxacin [11.0 (2011) to 4.9 (2024) DDD/1000 patient-days], cefepime [41.1 (2011) to 11.9 (2024) DDD/1000 patient-days], and ertapenem [13.8 (2011) to 7.5 (2024) DDD/1000 patient-days] (*p* < 0.05). On the contrary, there was a significant increase in the consumption of IV amoxicillin–clavulanate [32.9 (2011) to 48.5 (2024) DDD/1000 patient-days and piperacillin–tazobactam [47.8 (2011) to 82.0 (2024) DDD/1000 patient-days] (*p* < 0.05) [Table 2, Figure 2]. There were no significant changes in overall trends for ceftriaxone and meropenem consumption.

DDD expressed in median (IQR). Trend stated as increasing or decreasing is based on positive or negative *Kendall’s tau* coefficient, respectively, with corresponding statistical significance (*p* < 0.05). Trend stated as stable is based on no statistically significant monotonic trend (*p* ≥ 0.05).

In the initial phase, following hospital-wide PAF for carbapenems and IV ciprofloxacin/piperacillin–tazobactam in 2011 and 2013, respectively, there was no apparent reduction in the consumption of these antibiotics. Ironically, there was a noticeable decline in non-PAF antibiotics, where ceftriaxone and cefepime consumption reduced consistently from 105 to 74.8 DDD/1000 patient-days and 20.5 to 12.9 DDD patient-days, respectively, from 2011 to 2015. Interestingly, with the implementation of CDSS and the mandatory entry of antibiotic duration in 2015 and 2018, respectively, during the advancement phase, meropenem consumption reduced substantially—from 50.2 (2015) to 20.9 (2018) DDD per 1000 patient-days. This reduction was coupled with an apparent increase in IV co-amoxiclav and piperacillin–tazobactam consumption after 2015 [Figure 2].

During the optimisation/innovation phase, the ASP expanded its focus to narrower-spectrum antibiotics, including IV ciprofloxacin, IV co-amoxiclav, and ceftriaxone, which were often used inappropriately in extended surgical prophylaxis. Following this, we observed a modest reduction in IV ciprofloxacin, while IV co-amoxiclav and ceftriaxone consumption stabilised. The reduction in IV ciprofloxacin could also possibly be attributed to the concurrent decline in the susceptibility of *E. coli*, *Klebsiella* spp., and *Enterobacter* spp., rendering it less suitable for culture-directed therapy. Notably, there was a modest reduction in cefepime and ertapenem after 2019, which corresponds to an increase in meropenem consumption thereafter [Figure 2].

### 3.2. Antibiotic Appropriateness

**Figure 3:**
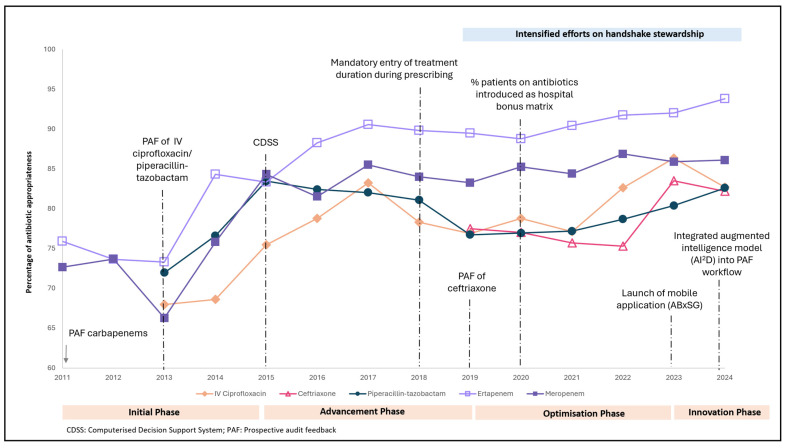
Appropriateness of PAF antibiotics (2011 to 2024).

Overall, there was significant improvement in the appropriateness trends of the following PAF antibiotics: 72% (2013) to 82.6% (2024) for IV ciprofloxacin, 75.9% (2011) to 93.8% (2024) for ertapenem, and 72.2% (2011) to 86.1% (2024) for meropenem (*p* < 0.05). Although not statistically significant, ceftriaxone and piperacillin–tazobactam appropriateness increased from 77.5% in 2019 to 82.2% in 2024 (*p* = 0.851) and from 67.9% in 2013 to 82.7% in 2024 (*p* = 0.411), respectively [Figure 3].

In the initiation phase, we observed a pronounced improvement in appropriateness of ertapenem [75.9% (2011) to 83.3% (2015)], meropenem [72.7% (2011) to 84.3% (2015)], and piperacillin–tazobactam [72.0% (2013) to 83.5% (2015)] following hospital-wide PAF. With CDSS implementation in 2015, during the advancement phase, there was an appreciable increase in IV ciprofloxacin [75.5% (2015) to 83.2% (2017)] and ertapenem [83.3% (2015) to 90.6% (2017)]. Of note, the CDSS provided no recommendations for the empiric use of ertapenem and only limited recommendations for IV ciprofloxacin, hence suggesting a possible shift away from the empiric use of IV ciprofloxacin/ertapenem to culture-directed prescribing.

During the optimisation/innovation phase, the appropriateness of carbapenems largely remained stable. This prompted the multi-pronged ASP strategies to shift focus from carbapenems to narrower-spectrum antibiotics, along with the expansion of ASP activities from infection management to surgical prophylaxis. Following this change in focus, we observed sustained improvements in the appropriate use of ceftriaxone [77.5% (2019) to 82.2% (2024)], IV ciprofloxacin [76.9% (2019) to 82.7% (2024)], and piperacillin–tazobactam [76.7% (2019) to 82.6% (2024)].

### 3.3. GNB Susceptibility

**Figure 4:**
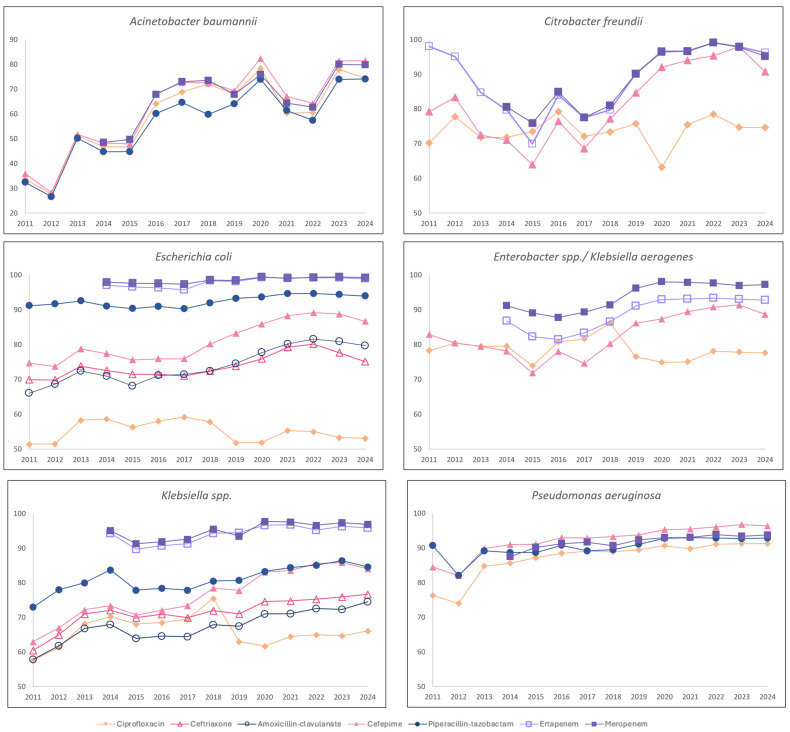
Hospital-wide susceptibility of 6 Gram-negative bacilli towards 7 antibiotics (from 2011 to 2024). Note: *Klebsiella* spp. excludes *Klebsiella aerogenes*. The *y*-axis for *A. baumannii* susceptibility data begins at 20% and for other Gram-negative bacilli starts at 50%.

From 2011 to 2024, the hospital-wide susceptibility trends of all six GNBs showed significant increases (*p* < 0.05), except for *E. coli*’s susceptibility to ertapenem and *Enterobacterales*’s susceptibility to ciprofloxacin [Table 3 and Figure 4].

*A. baumannii* and *P. aeruginosa*, both commonly associated with nosocomial infections, showed a significant increase in susceptibility to ciprofloxacin, cefepime, piperacillin–tazobactam, and meropenem over time (*p* < 0.05). Notably, the most rapid improvement in susceptibility was observed between 2011 and 2016, where ASP efforts were mainly focused on intensive PAF regarding carbapenems/anti-pseudomonal agents and CDSS implementation. The susceptibility of *E. coli* and *Klebsiella* spp. to ceftriaxone [often used as surrogate for resistance due to ESBL] and cefepime increased significantly (*p* < 0.05) from 2011 to 2024. *Enterobacter* spp./*Klebsiella aerogenes* and *Citrobacter freundii,* which are *Enterobacterales* that possesses *AmpC* β-lactamase enzymes, also showed improved susceptibility trends to cefepime, ertapenem, and meropenem (*p* < 0.05). The most rapid improvement in susceptibility of the *Enterobacterales* was observed after 2018, where it was preceded with a noticeable reduction in ceftriaxone and cefepime consumption from 2011 to 2016.

Interestingly, despite a marked increase in IV amoxicillin–clavulanate and piperacillin–tazobactam consumption, particularly after 2016, *E. coli* and *Klebsiella* spp. exhibited a pronounced increase in susceptibility to these antibiotics.

## 4. Discussion

To date, this is the first study to describe the evolution of antibiotic stewardship strategies over a 17-year period, and evaluate the trends of the volume/quality of antibiotic prescribing and susceptibility trends of commonly seen GNBs in a single quaternary healthcare institution. We demonstrated sustained improvement in appropriate prescribing of seven commonly used broad-spectrum IV antibiotics. Additionally, there was an overall reduction in the consumption of IV ciprofloxacin, cefepime, and ertapenem from 2011 to 2024 (*p* < 0.05), and a steep reduction in ceftriaxone consumption by approximately 30% in the initial phase of the ASP. Importantly, we demonstrated significant increases in hospital-wide susceptibility trends for all six commonly seen GNBs to all six β-lactam antibiotics (*p* < 0.05), except for *E. coli*’s susceptibility to ertapenem (which remained stable). Furthermore, the susceptibility of *A. baumannii* and *P. aeruginosa* to ciprofloxacin improved significantly over time (*p* < 0.05).

Reducing antibiotic consumption to mitigate antibiotic resistance is a core objective of ASPs [1,2]. In the initial and early advancement phase, ceftriaxone and cefepime consumption declined markedly by approximately one-third from 2011 to 2016, after which usage plateaued [Figure 2]. Although PAF targeted carbapenems and piperacillin–tazobactam rather than ceftriaxone at that time, recommendations for antibiotic cessation when therapy was no longer indicated likely influenced overall prescribing behaviour. This contrasted with pre-PAF practices, where clinicians often preferred antibiotic de-escalation instead of discontinuation. The combination of PAF and the rolling out of institutional antibiotic guidelines likely fostered a behavioural shift among prescribers, promoting greater awareness and adherence to the judicious use of narrower-spectrum antibiotics.

Interestingly, reductions in cephalosporin consumption coincided with pronounced improvements in *A. baumannii* susceptibility from 2011 to 2016, with susceptibility rates towards all tested antibiotics nearly doubling during this period. This improvement is consistent with previous evidence linking ceftriaxone use to the selection of multidrug-resistant *A. baumannii* [24]. A subsequent improvement in *Enterobacterales* susceptibility to ceftriaxone (reflecting declining ESBL prevalence) and cefepime occurred from 2018 onwards, albeit after a lag of >3 years following reduced cephalosporin use—an ecological delay similarly reported by Livermore et al. [25]. The persistence of resistance despite reduced selective pressure may be attributable to the efficient horizontal transfer of ESBL-encoding plasmids through conjugation, maintaining resistance within bacterial populations [26]. Furthermore, we postulate that the decline in IV ciprofloxacin use from 2019 contributed to the sustained reduction in ESBL prevalence [27], likely driven by ASP initiatives targeting ciprofloxacin use in surgical prophylaxis and the known reduced susceptibility of *Enterobacterales* to ciprofloxacin nationally [28]. On the contrary, our data showed improved ciprofloxacin susceptibility by about 15% and 40% in hospital-acquired pathogens such as *P. aeruginosa* and *A. baumannii*, respectively from 2011 to 2024, supporting the judicious use of ciprofloxacin within the institution for nosocomial infections [29].

Unlike cephalosporins and ciprofloxacin, an association between antibiotic consumption and GNB susceptibility rates was not consistently observed across all antibiotics in our study. Despite the increased consumption of almost 50% and 70% of IV amoxicillin–clavulanate and piperacillin–tazobactam, respectively, from 2011 to 2024 (*p* < 0.05), a trend similarly observed nationwide [28], there was significant improvement in GNB susceptibility to amoxicillin–clavulanate and piperacillin–tazobactam over time (*p* < 0.05). Similarly, Lee et al. observed that increased piperacillin–tazobactam utilisation did not correspond with higher resistance rates in *E. coli*, suggesting a higher threshold for resistance acquisition [30]. The same study also found no rise in *AmpC-*β-lactamase producing *E. coli* or *K. pneumoniae*. Additionally, Marquet et al. reported that amoxicillin–clavulanate use was protective against third-generation cephalosporin non-susceptibility in *K. pneumoniae*, supporting its role as a potential alternative to cephalosporins or fluoroquinolones to mitigate resistance [31].

In practice, some degree of the “balloon-squeezing” effect is unavoidable, particularly in quaternary healthcare settings where managing complex and critically ill patients is the norm. At our institution, a reduction in meropenem consumption following the implementation of the CDSS in 2015 was accompanied by an increased use of IV amoxicillin–clavulanate and piperacillin–tazobactam, likely indicating the effectiveness of the CDSS in assisting doctors in choosing narrower-spectrum antibiotics as empiric therapy. Therefore, ASPs should avoid self-penalisation based solely on antibiotic usage matrices, recognising that the rise in the consumption of selected antibiotics may be deemed clinically reasonable within acceptable boundaries.

Similarly, following 2019, meropenem consumption increased slowly along with a further reduction in cefepime and ertapenem use. The rise in meropenem use was likely influenced by emerging evidence and local experience of cefepime/ertapenem-induced neurotoxicity [32,33], which led the ASP team to permit meropenem for infections caused by *AmpC*- or ESBL-producing *Enterobacterales* in patients with severe renal impairment. Additionally, the incorporation of evidence supporting higher β-lactam dosing in critically ill or obese patients into our institutional antibiotic guidelines has likely contributed to the increased consumption of not only meropenem, but also piperacillin–tazobactam [34,35]. In 2023, β-lactam therapeutic drug monitoring (TDM) was introduced to optimise pharmacokinetic/pharmacodynamic targets with the aim of both improving patient outcomes and suppressing the emergence of antibiotic resistance [17,18]. Although this coincided with a slight increase in the consumption of piperacillin–tazobactam and meropenem in 2023–2024, no deterioration in GNB susceptibility was observed in 2024.

Antibiotic consumption trends may not always align with appropriate prescribing trends, and effective antibiotic stewardship requires balancing the evaluation of both indicators. A major achievement of our 17-year ASP has been the sustained improvement in appropriate antibiotic prescribing across all antibiotics evaluated in our study. PAF, which was our cornerstone ASP strategy during the initial phase, has led to improved appropriateness in IV ciprofloxacin, piperacillin–tazobactam, and carbapenem administration. Interestingly, it took 2 years of PAF before carbapenem appropriateness improved. This delayed improvement suggests that building prescriber rapport through ongoing handshake stewardship requires time. Compared to restrictive measures, enabling strategies like PAF allows real-time feedback to be provided to prescribers, which provides opportunities to engage, educate, and empower [36].

A coordinated approach with strong senior leadership support is critical for the success and sustainability of ASPs [2]. The provision of financial and manpower resources to develop technology enablers during the optimisation/innovation phase—including the CDSS, which had driven improvements in carbapenem appropriateness in our study—was also proven effective in other healthcare settings [37,38]. As our ASP has matured, real-time surveillance dashboards and analytics have enabled the team to promptly identify prescribing gaps in narrower-spectrum antibiotics. For example, several surgical departments were found to use unnecessarily prolonged antibiotics for surgical prophylaxis. In response, targeted strategies were implemented, including the expansion of PAF to ceftriaxone in surgical departments and collaborations with high-usage teams to optimise antibiotic duration [14]. Furthermore, the launch of an in-house antibiotic mobile application (ABxSg) in 2023, which provides physicians with on-the-go access to antibiotic prescribing guidelines and resources, was designed to promote appropriate antibiotic use across all antibiotics. This strategy has reduced the proportion of hospitalised patients prescribed antibiotics in our institution [15]. Additionally, substantial financial investment supported the development of an augmented intelligence model (AI^2^D), which had an accuracy of 80% in predicting the likelihood of acute bacterial lower respiratory tract infection. The integration of AI^2^D into PAF enhanced its effectiveness; antibiotics prescribed to such patients were nearly four times more likely to be discontinued within two days compared with cases managed without AI^2^D [16].

During the advancement phase, significant efforts were directed towards concurrent upskilling of the workforce, including supporting clinical pharmacists through national residency programmes and relevant postgraduate studies. Clinically advanced pharmacists are then better equipped to manage complex patients and lead advanced ASP strategies, which strengthens the identity and visibility of the ASP team within the hospital. Beyond participating in ward rounds, ASP pharmacists and physicians were actively involved in multiple quality improvement projects, including projects pertaining to diagnostic stewardship with major departments as part of handshake stewardship initiatives. The consistent presence and collaboration of a capable ASP team positioned the team as trusted partners in patient care rather than mere “enforcers” of appropriate antibiotic use. This branding improved rapport with prescribers, making them more receptive to ASP recommendations and strategies, thereby contributing to sustained improvements in antibiotic prescribing appropriateness. Notably, appropriate ertapenem and meropenem prescribing increased substantially from 75.9% (2011) to 93.8% (2024) and from 72.7% (2011) to 86.1% (2024), respectively. These results represent an exceptional achievement when compared with reports from hospitals in other parts of Asia, Europe, and North America, where carbapenem appropriateness rates typically range between 50% and 60% [39,40,41].

### Study Limitations

Firstly, in the initial phase, the hospital’s electronic system lacked the functionality to capture several critical data types, limiting the ability to comprehensively evaluate the impact of early ASP strategies. For example, complete antibiotic consumption/appropriateness data and carbapenem susceptibility data were not available before 2011 and 2014, respectively. Furthermore, hospital-wide consumption trends could not be tracked for all antibiotics. For instance, the accurate monitoring of narrow-spectrum antibiotics, such as IV cefazolin commonly used for surgical prophylaxis at operating theatres, was not feasible due to limitations in the current electronic database capabilities. Secondly, we are unable to distinguish between clinical isolates originating from community-acquired and hospital-acquired infections. Improved susceptibility trends may possibly reflect increased awareness of antibiotic stewardship among community prescribers over time; however, causality cannot be accounted for in our study. This highlights the need to enhance the capabilities of our analytical dashboards to allow segregation according to infection origin. Thirdly, although enhanced infection control measures were progressively implemented during the study period and likely mitigated the transmission-resistant GNBs, these effects could not be incorporated into our analysis. Such measures included intensified strategies to reduce hospital-acquired infection risk, expanded surveillance for drug-resistant GNBs, improved hand hygiene through targeted campaigns and compliance audits, and enhanced environmental cleaning using advanced disinfection technologies [42]. Lastly, the design of the study limits causal inference, as the trend analysis was primarily intended to explore whether monotonic trends occurred over time with the evolution of ASP strategies.

## 5. Conclusions

During the 17-year period in which a multi-pronged ASP approach was implemented, improvements were observed in antibiotic prescribing and in GNB susceptibility to most antibiotics at our institution. Beyond PAF, technology enablers and handshake stewardship have further advanced our efforts and yielded measurable results. Equally important is strengthening the visibility and branding of the ASP, with sustained support from hospital leadership, which is critical for long-term success. In a rapidly evolving healthcare landscape, ASPs must remain agile, continually refining priorities and employing innovative strategies. Incorporating diagnostic stewardship alongside advances in rapid diagnostics, immunoprofiling, and machine learning can further enhance the judicious use of antibiotics [43,44,45,46,47].

## Figures and Tables

**Table 1:** Four phases of the antibiotic stewardship programme and corresponding key strategies.

Phase	Years	Key Stewardship Strategies
Initiation	2008–2014	Development of in-house antibiotic guidelines Implementing prospective audit feedback (PAF) ○ 2008–2011: PAF of meropenem, ertapenem, piperacillin tazobactam from selected departments ○ 2011 onwards: Expansion to hospital-wide PAF of meropenem and ertapenem ○ 2013 onwards: Expansion to hospital-wide PAF of piperacillin-tazobactam and IV ciprofloxacin Feedback to physicians; initiation of handshake stewardship ○ Feedback reports sent to head of departments to improve appropriate prescribing every 3 months
Advancement	2015–2018	Harnessing technology to improve antibiotic prescribing ○ 2015: Hospital-wide computerised decision support system ○ 2018: Mandatory entry of antibiotic duration for select broad-spectrum antibiotics upon prescribing Enhancing identity and visibility of ASP team ○ Pharmacists participated in ward rounds with departments identified to have high antibiotic use ○ Electronic documentation of PAF interventions Rapid expansion of training of highly competent clinical pharmacists with expertise in infectious diseases ○ Trainings include national residency programmes and relevant postgraduate studies
Optimisation	2019–2023	Garnering support from senior management ○ Incorporating proportion of hospitalised patients prescribed antibiotics as bonus matrix in 2020 Strengthening analytics capabilities ○ Building of real-time dashboards and reports to facilitate surveillance of antibiotic use and identification of prescribing gaps Expansion of ASP focus to include narrower-spectrum antibiotics ○ Expansion of focus beyond antibiotics used for treatment to surgical prophylaxis ○ Expansion of PAF to include IV ceftriaxone use in surgical departments in 2019 Reinforcing handshake stewardship ○ Enhanced collaboration through quality improvement projects with major surgical departments ○ Conducting regular roadshow to major departments to promote appropriate antibiotic prescribing Enhancing identity and visibility of ASP team by leveraging corporate social media ○ Leveraged corporate social media to showcase endorsements of ASP initiatives by hospital leaders and senior clinicians ○ Recognition and awards presented to physicians acknowledged as ASP champions ○ Promoted best practices pertaining to appropriate antibiotic use
Innovation	2023–date	Launched antibiotic mobile application (ABxSG) Initiated therapeutic drug monitoring of β-lactams Integrated augmented intelligence model (AI^2^D) into PAF workflow to improve workflow efficiency Collaborated with select departments to improve diagnostic stewardship on ordering of urine cultures

**Table 2:** Defined daily doses per 1000 patient-days of 7 commonly used antibiotics (2011 to 2024).

Antibiotic	DDD/1000 PD (2011)	DDD/1000 PD (2024)	DDD	Trend (2011–2024)	*Kendall tau* Coefficient	*p*-Value
IV Ciprofloxacin	11.0	4.9	7.8 (4.4–9.4)	Decreasing	−0.853	0.000
Ceftriaxone	105.0	90.0	77.2 (73.7–82.9)	Stable	−0.165	0.412
IV Amoxicillin–clavulanate	32.9	48.5	39.8 (34.2–43.4)	Increasing	0.780	0.000
Cefepime	41.1	11.9	25.8 (12.8–30.2)	Decreasing	−0.641	0.001
Piperacillin-tazobactam	47.8	82.0	70.9 (63.8–78.5)	Increasing	0.780	0.000
Ertapenem	13.8	7.5	9.0 (7.7–10.2)	Decreasing	−0.751	0.000
Meropenem	38.3	47.8	40.3 (38.3–44.7)	Stable	−0.011	0.956

DDD: defined daily dose; IV: intravenous; PD: patient days.

**Table 3:** Hospital-wide susceptibility trend of the 6 Gram-negative bacilli towards 7 antibiotics (from 2011 to 2024).

Gram- Negative Bacilli	No. of Isolates a Year ^◊^	Antibiotic	% Susceptibility * (as of 2011)	% Susceptibility (as of 2024)	Susceptibility Trend **	*Kendall’s tau* Coefficient	*p*-Value
*Acinetobacter* *baumannii*	254 (216–378)	Ciprofloxacin	33.6	74.4	Increasing	0.58	<0.01
		Cefepime	35.9	81.5	Increasing	0.56	<0.01
		Piperacillin– tazobactam	32.4	74.1	Increasing	0.66	<0.01
		Meropenem	48.5	79.8	Increasing	0.48	<0.05
*Citrobacter* *freundii*	99 (88–110)	Ciprofloxacin	70.2	74.6	stable	0.22	0.273
		Cefepime	79.2	90.7	Increasing	0.53	<0.05
		Ertapenem	79.7	96.2	Increasing	0.65	<0.01
		Meropenem	80.6	99.2	Increasing	0.67	<0.01
*Enterobacter* spp. (including *Klebsiella* *aerogenes)*	805 (747–885)	Ciprofloxacin	78.2	77.6	Stable	−0.17	0.412
		Cefepime	82.8	88.6	Increasing	0.47	<0.05
		Ertapenem	86.7	92.7	Increasing	0.60	<0.05
		Meropenem	91.1	97.2	Increasing	0.52	<0.05
*E. Coli*	4282 (3924–4738)	Ciprofloxacin	51.4	53.1	Stable	−0.10	0.622
		Ceftriaxone	69.9	75.1	Increasing	0.59	<0.01
		Amoxicillin–clavulanate	66.0	79.6	Increasing	0.77	<0.01
		Cefepime	74.7	86.6	Increasing	0.73	<0.01
		Piperacillin– tazobactam	91.1	93.9	Increasing	0.49	<0.05
		Ertapenem	97.0	98.9	Stable	0.45	0.059
		Meropenem	97.9	99.2	Increasing	0.55	<0.05
*Klebsiella* spp.	2323 (2180–2767)	Ciprofloxacin	57.6	66.1	Stable	0.08	0.702
		Ceftriaxone	60.5	76.7	Increasing	0.77	<0.01
		Amoxicillin–clavulanate	57.9	74.5	Increasing	0.76	<0.01
		Cefepime	63.0	84.1	Increasing	0.84	<0.01
		Piperacillin– tazobactam	73.0	84.6	Increasing	0.69	<0.01
		Ertapenem	94.3	95.9	Increasing	0.62	<0.01
		Meropenem	95.1	96.9	Increasing	0.53	<0.05
*Pseudomonas* *aeruginosa*	1522 (1380–1831)	Ciprofloxacin	76.3	91.3	Increasing	0.93	<0.01
		Cefepime	84.5	96.4	Increasing	0.93	<0.01
		Piperacillin– tazobactam	90.7	92.9	Increasing	0.65	<0.01
		Meropenem	87.5	93.8	Increasing	0.86	<0.01

**^◊^** No. of isolates are presented as median (interquartile range). * 2014 onwards for ertapenem and meropenem. ** Susceptibility trend stated as increasing or decreasing is based on statistically significant positive or negative *Kendall’s tau* coefficient, respectively (*p* < 0.05). Susceptibility trend stated as stable is based on no statistically significant monotonic trend (*p* ≥ 0.05).

## Data Availability

The original contributions presented in this study are included in the article.

## References

[1] Barlam T.F., Cosgrove S.E., Abbo L.M., MacDougall C., Schuetz A.N., Septimus E.J., Srinivasan A., Dellit T.H., Falck-Ytter Y.T., Fishman N.O. (2016). Implementing an Antibiotic Stewardship Program: Guidelines by the Infectious Diseases Society of America and the Society for Healthcare Epidemiology of America. Clin. Infect. Dis..

[2] Pollack L.A., Srinivasan A. (2014). Core elements of hospital antibiotic stewardship programs from the Centers for Disease Control and Prevention. Clin. Infect. Dis..

[3] Chua A.Q., Kwa A., Tan T.Y., Legido-Quigley H., Hsu L.Y. (2019). Ten-year narrative review on antimicrobial resistance in Singapore. Singap. Med. J..

[4] Ng T.M., Ang L.W., Heng S.T., Kwa A.L.-H., Wu J.E., Seah X.F.V., Lee S.Y., Seah J., Choo R., Lim P.L. (2023). Antibiotic utilisation and resistance over the first decade of nationally funded antimicrobial stewardship programmes in Singapore acute-care hospitals. Antimicrob. Resist. Infect. Control..

[5] Ababneh M.A., Nasser S.A., Rababa’h A.M. (2021). A systematic review of Antimicrobial Stewardship Program implementation in Middle Eastern countries. Int. J. Infect. Dis..

[6] Karanika S., Paudel S., Grigoras C., Kalbasi A., Mylonakis E. (2016). Systematic Review and Meta-analysis of Clinical and Economic Outcomes from the Implementation of Hospital-Based Antimicrobial Stewardship Programs. Antimicrob. Agents Chemother..

[7] Strazzulla A., Adrien V., Houngnandan S.R., Devatine S., Bahmed O., Abroug S., Hamrouni S., Monchi M., Diamantis S. (2024). Characteristics of Pseudomonas aeruginosa infection in intensive care unit before (2007–2010) and after (2011–2014) the beginning of an antimicrobial stewardship program. Antimicrob. Steward. Healthc. Epidemiol..

[8] Mahmoudi L., Sepasian A., Firouzabadi D., Akbari A. (2020). The Impact of an Antibiotic Stewardship Program on the Consumption of Specific Antimicrobials and Their Cost Burden: A Hospital-wide Intervention. Risk Manag. Healthc. Policy.

[9] Chrysou K., Zarkotou O., Kalofolia S., Papagiannakopoulou P., Mamali V., Chrysos G., Themeli-Digalaki K., Sypsas N., Tsakris A., Pournaras S. (2022). Impact of a 4-year antimicrobial stewardship program implemented in a Greek tertiary hospital. Eur. J. Clin. Microbiol. Infect. Dis..

[10] Liew Y.X., Lee W., Loh J.C.Z., Cai Y., Tang S.S.L., Lim C.L.L., Teo J., Ong R.W.Q., Kwa A.L.-H., Chlebicki M.P. (2012). Impact of an antimicrobial stewardship programme on patient safety in Singapore General Hospital. Int. J. Antimicrob. Agents.

[11] Loo L.W., Liew Y.X., Lee W., Lee L.W., Chlebicki P., Kwa A.L.-H. (2019). Discontinuation of antibiotic therapy within 24 hours of treatment initiation for patients with no clinical evidence of bacterial infection: A 5-year safety and outcome study from Singapore General Hospital Antimicrobial Stewardship Program. Int. J. Antimicrob. Agents.

[12] Teo J., Kwa A.L.H., Loh J., Chlebicki M.P., Lee W. (2012). The effect of a whole-system approach in an antimicrobial stewardship programme at the Singapore General Hospital. Eur. J. Clin. Microbiol. Infect. Dis..

[13] Versporten A., Zarb P., Caniaux I., Gros M.-F., Drapier N., Miller M., Jarlier V., Nathwani D., Goossens H., Koraqi A. (2018). Antimicrobial consumption and resistance in adult hospital inpatients in 53 countries: Results of an internet-based global point prevalence survey. Lancet Glob. Health.

[14] Loo L.W., Zhou Y.P., Wang Y.B., Lee L.W., Chung J.S. (2025). Antimicrobial Stewardship in Cardiac Device Surgery: Impact of Behavioural Change Interventions on Extended Prophylaxis Practices. Antibiotics.

[15] Lee L.W., Lim S.Y.C., Zhou Y.P., Chung S.J., Chin D.Z., Kwa A.L.H., Lee W.H.L. (2025). Impact of the ABxSG Mobile Application on Antibiotic Prescribing: An Interrupted Time Series Study. Antibiotics.

[16] Tang S., Lim J.L., Lee L.X.T., Yii Y.C.D., Zhou Y.P., Wang Y., Cherng P.Z.B., Chlebicki P.M., Gan L.S.C., Thien S.Y. Augmented Intelligence in Infectious Diseases (AI^2^D) as an Antimicrobial Stewardship Tool for Early Antibiotic Discontinuation in Suspected Lower Respiratory Tract Infections [Abstract]. Proceedings of the Congress of the European Society of Clinical Microbiology and Infectious Diseases (ESCMID).

[17] Sumi C.D., Heffernan A.J., Lipman J., Roberts J.A., Sime F.B. (2019). What Antibiotic Exposures Are Required to Suppress the Emergence of Resistance for Gram-Negative Bacteria? A systematic review. Clin. Pharmacokinet..

[18] Roberts J.A., Paul S.K., Akova M., Bassetti M., De Waele J.J., Dimopoulos G., Kaukonen K.-M., Koulenti D., Martin C., Montravers P. (2014). DALI: Defining antibiotic levels in intensive care unit patients: Are current β-lactam antibiotic doses Sufficient for critically ill patients?. Clin. Infect. Dis..

[19] WHO Collaborating Centre for Drug Statistics Methodology (2024). ATC Classification Index with DDDs, 2024.

[20] CLSI (2022). Analysis and Presentation of Cumulative Antimicrobial Susceptibility Test Data.

[21] CLSI (2024). Piperacillin-Tazobactam Breakpoints for Pseudomonas aeruginosa.

[22] Tamma P.D., A Harris P.N., Mathers A.J., Wenzler E., Humphries R.M. (2023). Breaking Down the Breakpoints: Rationale for the 2022 Clinical and Laboratory Standards Institute Revised Piperacillin-Tazobactam Breakpoints Against Enterobacterales. Clin. Infect. Dis..

[23] Bork J.T., Heil E.L., Leekha S., Fowler R.C., Hanson N.D., Majumdar A., Johnson J.K. (2017). Impact of CLSI and EUCAST Cefepime breakpoint changes on the susceptibility reporting for Enterobacteriaceae. Diagn. Microbiol. Infect. Dis..

[24] Mihalov P., Hodosy J., Koščálová A., Čaprnda M., Kachlíková M., Jurenka J., Bendžala M., Sabaka P. (2023). Antimicrobial Therapy as a Risk Factor of Multidrug-Resistant Acinetobacter Infection in COVID-19 Patients Admitted to the Intensive Care Unit. Can. J. Infect. Dis. Med. Microbiol..

[25] Livermore D.M., Hope R., Reynolds R., Blackburn R., Johnson A.P., Woodford N. (2013). Declining cephalosporin and fluoroquinolone non-susceptibility among bloodstream Enterobacteriaceae from the UK: Links to prescribing change?. J. Antimicrob. Chemother..

[26] Moosdeen F. (1997). The evolution of resistance to cephalosporins. Clin. Infect. Dis..

[27] Aldeyab M.A., Harbarth S., Vernaz N., Kearney M.P., Scott M.G., Elhajji F.W.D., Aldiab M.A., McElnay J.C. (2012). The impact of antibiotic use on the incidence and resistance pattern of extended-spectrum beta-lactamase-producing bacteria in primary and secondary healthcare settings. Br. J. Clin. Pharmacol..

[28] Ministry of Health (2019). One Health Report on Antimicrobial Utilisation and Resistance 2019.

[29] Medina Presentado J.C., Paciel López D., Berro Castiglioni M., Gerez J. (2011). Ceftriaxone and ciprofloxacin restriction in an intensive care unit: Less incidence of *Acinetobacter* spp. and improved susceptibility of *Pseudomonas aeruginosa*. Rev. Panam. Salud Publica.

[30] Lee J., Oh C.E., Choi E.H., Lee H.J. (2013). The impact of the increased use of piperacillin/tazobactam on the selection of antibiotic resistance among invasive Escherichia coli and Klebsiella pneumoniae isolates. Int. J. Infect. Dis..

[31] Marquet A., Vibet M.-A., Caillon J., Javaudin F., Chapelet G., Montassier E., Batard E. (2018). Is There an Association Between Use of Amoxicillin-Clavulanate and Resistance to Third-Generation Cephalosporins in Klebsiella pneumoniae and Escherichia coli at the Hospital Level?. Microb. Drug Resist..

[32] Payne L.E., Gagnon D.J., Riker R.R., Seder D.B., Glisic E.K., Morris J.G., Fraser G.L. (2017). Cefepime-induced neurotoxicity: A systematic review. Crit. Care.

[33] Wang C., Zhou Y., Zhou Y., Ye C. (2023). Ertapenem-Induced Neurotoxicity: A Literature Review of Clinical Characteristics and Treatment Outcomes. Infect. Drug Resist..

[34] Alobaid A.S., Wallis S.C., Jarrett P., Starr T., Stuart J., Lassig-Smith M., Mejia J.L., Roberts M.S., Roger C., Udy A.A. (2017). Population Pharmacokinetics of Piperacillin in Nonobese, Obese, and Morbidly Obese Critically Ill Patients. Antimicrob. Agents Chemother..

[35] (2022). European Committee on Antimicrobial Susceptibility Testing. Aminopenicillin Breakpoints for Enterobacterales. General Consultation. https://www.eucast.org/fileadmin/src/media/PDFs/EUCAST_files/Consultation/2021/Aminopenicillins_and_Enterobacterales_General_consultation_November_2021.pdf.

[36] Davey P., A Marwick C., Scott C.L., Charani E., McNeil K., Brown E., Gould I.M., Ramsay C.R., Michie S. (2017). Interventions to improve antibiotic prescribing practices for hospital inpatients. Cochrane Database Syst. Rev..

[37] Nachtigall I., Tafelski S., Deja M., Halle E., Grebe M.C., Tamarkin A., Rothbart A., Uhrig A., Meyer E., Musial-Bright L. (2014). Long-term effect of computer-assisted decision support for antibiotic treatment in critically ill patients: A prospective ‘before/after’ cohort study. BMJ Open.

[38] Paul M., Andreassen S., Tacconelli E., Nielsen A.D., Almanasreh N., Frank U., Cauda R., Leibovici L., TREAT Study Group (2006). Improving empirical antibiotic treatment using TREAT, a computerized decision support system: Cluster randomized trial. J. Antimicrob. Chemother..

[39] Poline J., Postaire M., Parize P., Pilmis B., Bille E., Zahar J.R., Frange P., Cohen J.F., Lortholary O., Toubiana J. (2021). Stewardship program on carbapenem prescriptions in a tertiary hospital for adults and children in France: A cohort study. Eur. J. Clin. Microbiol. Infect. Dis..

[40] Ishtiaq U., Acosta K., Akabusi C., Noble K., Gujadhur N., Cluzet V. (2024). Appropriateness of Empiric Initiation of Meropenem in the Intensive Care Unit as Determined by Internal Medicine Residents. Antimicrob. Steward. Healthc. Epidemiol..

[41] Zhang D., Cui K., Lu W., Bai H., Zhai Y., Hu S., Li H., Dong H., Feng W., Dong Y. (2019). Evaluation of carbapenem use in a tertiary hospital: Antimicrobial stewardship urgently needed. Antimicrob. Resist. Infect. Control..

[42] National Infection Prevention and Control Committee (2022). The National Infection Prevention and Control Standards for Acute Healthcare Facilities.

[43] Zakhour J., Haddad S.F., Kerbage A., Wertheim H., Tattevin P., Voss A., Ünal S., Ouedraogo A.S., Kanj S.S. (2023). International Society of Antimicrobial Chemotherapy (ISAC) and the Alliance for the Prudent Use of Antibiotics (APUA). Diagnostic stewardship in infectious diseases: A continuum of antimicrobial stewardship in the fight against antimicrobial resistance. Int. J. Antimicrob. Agents.

[44] Claeys K.C., Trautner B.W., Leekha S., Coffey K.C., Crnich C.J., Diekema D.J., Fakih M.G., Goetz M.B., Gupta K., Jones M.M. (2022). Optimal Urine Culture Diagnostic Stewardship Practice-Results from an Expert Modified-Delphi Procedure. Clin. Infect. Dis..

[45] Pinto-de-Sá R., Sousa-Pinto B., Costa-de-Oliveira S. (2024). Brave New World of Artificial Intelligence: Its Use in Antimicrobial Stewardship—A Systematic Review. Antibiotics.

[46] Tang S., Chang D., Zhi Chin D., Piotr Chlebicki M., Jasmine Chung S., Wei Lee L., Lee W., Zhou P.Y., Kwa A. (2023). 163. Can Machine Learning Guide Antibiotic Initiation for Lower Respiratory Tract Infections?. Open Forum Infect. Dis..

[47] E Hanson K., Tsalik E.L. (2025). Host Immune Response Profiling for the Diagnosis of Infectious Diseases. J. Infect. Dis..

